# Note on Quarantine

**Published:** 1862-04

**Authors:** 


### NOTE ON QUARANTINE.

In the article on the Report of the Social Science Association Committee on Quarantine in our last number, it was erroneously stated that Dr. Bryson had not signed the Report. The author of the article, preoccupied with Dr. Bryson's dissentient opinions, had overlooked his name among the signatures attached to the Report, until his attention was called to the omission, of which he had unintentionally been guilty.

